# The Influence of Oncogenic RAS on Chemotherapy and Radiotherapy Resistance Through DNA Repair Pathways

**DOI:** 10.3389/fcell.2022.751367

**Published:** 2022-03-11

**Authors:** Rodrigo E. Cáceres-Gutiérrez, Yair Alfaro-Mora, Marco A. Andonegui, José Díaz-Chávez, Luis A. Herrera

**Affiliations:** ^1^ Unidad de Investigación Biomédica en Cáncer, Instituto Nacional de Cancerología-Instituto de Investigaciones Biomédicas, UNAM, Mexico City, Mexico; ^2^ Instituto Nacional de Medicina Genómica, Mexico City, Mexico

**Keywords:** ras, oncogene-induced senescence, reactive oxygen species, DNA damage response, double strand breaks, cancer, chemotherapy and radiotherapy resistance

## Abstract

RAS oncogenes are chief tumorigenic drivers, and their mutation constitutes a universal predictor of poor outcome and treatment resistance. Despite more than 30 years of intensive research since the identification of the first RAS mutation, most attempts to therapeutically target RAS mutants have failed to reach the clinic. In fact, the first mutant RAS inhibitor, Sotorasib, was only approved by the FDA until 2021. However, since Sotorasib targets the KRAS G12C mutant with high specificity, relatively few patients will benefit from this therapy. On the other hand, indirect approaches to inhibit the RAS pathway have revealed very intricate cascades involving feedback loops impossible to overcome with currently available therapies. Some of these mechanisms play different roles along the multistep carcinogenic process. For instance, although mutant RAS increases replicative, metabolic and oxidative stress, adaptive responses alleviate these conditions to preserve cellular survival and avoid the onset of oncogene-induced senescence during tumorigenesis. The resulting rewiring of cellular mechanisms involves the DNA damage response and pathways associated with oxidative stress, which are co-opted by cancer cells to promote survival, proliferation, and chemo- and radioresistance. Nonetheless, these systems become so crucial to cancer cells that they can be exploited as specific tumor vulnerabilities. Here, we discuss key aspects of RAS biology and detail some of the mechanisms that mediate chemo- and radiotherapy resistance of mutant RAS cancers through the DNA repair pathways. We also discuss recent progress in therapeutic RAS targeting and propose future directions for the field.

## The RAS Oncogenes

The Ras superfamily is composed of structurally and mechanistically related small GTPase proteins organized in five major families named Ras, Rho, Arf, Ran, and Rab. In humans, the Ras family (20–29 kDa) encompasses 36 members of which KRAS, HRAS, NRAS, ERAS, RRAS, and MRAS are the archetypal elements (Rojas et al., 2012).

The main role of RAS proteins is the transduction of external stimuli into intracellular signaling cascades. These GTPases work as intracellular membrane-associated binary switches that trigger a broad range of cell survival and proliferation events. These proteins cycle around active and inactive states through their intrinsic GTPase activity and their interaction with Guanine Nucleotide Exchange Factors (GEFs) and GTPase-Activating Proteins (GAPS), which promote the GTP-bound active, and GDP-bound inactive states, respectively (Simanshu et al., 2017).

Structurally, RAS proteins bear a G domain that binds and hydrolyzes guanine nucleotides, and two loops (switch 1 and switch 2) that drive the conformational changes that facilitate the binding of effectors, exchange factors, and activators. The C terminal region of RAS (25 amino acids) contains a hypervariable region (HVR) which is poorly conserved among the Ras family members. This HVR is targeted by several pos-translational modifications and is crucial for insertion into and interaction with the plasma membrane (Prior and Hancock, 2001; Vetter and Wittinghofer, 2001; Hancock, 2003).

RAS activation relies on a plethora of membrane-associated receptors, like tyrosine kinase receptors, G-protein coupled receptors, integrins, or toll-like receptors (Cattaneo et al., 2014). When such receptors become activated by binding of their corresponding ligand, they recruit adaptor proteins and Guanine Exchange Factors (GEFs), which exchange RAS-associated GDP for GTP, thereby generating a conformational change in switch 1 and switch 2 loops. This conformational change exposes the residues necessary for RAS’ interaction with its downstream effectors, including Y40 for PI3K, E37 for Ral-GEF, and T35 for RAF (Schlessinger, 2000; Shields et al., 2000; Vetter and Wittinghofer, 2001). RAS activity generates transient downstream signaling cascades that activate several effectors like the Raf/Mek/Erk, PI3K/Akt, RalGDS/Ral, and Mekk/Sek/Jnk pathways, which regulate multiple cellular events through gene transcription, among other mechanisms. Although RAS’ GTP hydrolysis rate is intrinsically slow, its catalytic activity is importantly accelerated upon interaction with GTPase Activating Proteins (GAPs). GAPs can increase RAS GTPase activity about 10^5^-fold by inserting an arginine finger into RAS’ GTPase cleft. GTP hydrolysis leads RAS back to its inactive state (Drugan et al., 2000; Vetter and Wittinghofer, 2001; Pamonsinlapatham et al., 2009; Scheffzek and Shivalingaiah, 2019). In fact, the lifetime of active GTP-bound RAS is governed by the time of encounter with a GAP. Therefore, RAS-GAP inactivation or mutation, as well as RAS constitutive activation by inhibition of its GTP hydrolysis capacity, promotes sustained RAS signaling, which can ultimately lead to malignant transformation (Jett and Friedman, 2010).

The human RAS homologues of Harvey rat sarcoma viral oncogene (HRAS), Kirsten rat sarcoma viral oncogene (KRAS), and the neuroblastoma RAS viral oncogene (NRAS) become major disease drivers upon mutation: between 17 and 30% of all human tumors bear RAS mutations. Of these cancer-associated alterations, ∼97% occur in codons 12, 13, and 61 of the distinct isoforms (Simanshu et al., 2017; Mo et al., 2018). More specifically, these mutations are present in 50% of colon cancer cases (Logsdon and Lu, 2016) and ∼95% of pancreatic cancer cases, and are estimated to cause one million deaths per year worldwide (Simanshu et al., 2017). In addition, KRAS alterations are more frequently observed in lung, pancreatic, and colorectal malignancies, and NRAS mutations are present in hematological malignancies, while HRAS mutations are present in dermatological and head and neck malignancies (Pylayeva-Gupta et al., 2011). Overall, mutations in KRAS are the most common, accounting for ∼85% of all RAS mutations, followed by 12% for NRAS, and 3% for HRAS (Simanshu et al., 2017).

These alterations lead to critical amino acid substitutions which generate a constitutively active RAS protein, due to the impairment of GAP binding or decreased GTP hydrolysis (Smith et al., 2013). KRAS, NRAS, and HRAS have different mutation frequencies among each of the mutational hotspots. The predominant point of mutations in KRAS is G12 (89%), followed by G13 (9%), and to a lesser extent, Q61 (1%). However, in NRAS, Q61 is the most commonly mutated hotspot (60%), followed by G12 (25%), and G13 (14%). For HRAS, the most prevalent mutation is G12 (55%), followed by Q61 (36%), and then G13 (8%) (Prior et al., 2012; Hobbs et al., 2016; Lu et al., 2016).

The RAS mutations mentioned above confer oncogenic properties to the cell, like uncontrolled proliferation, loss of contact inhibition, increased motility, altered metabolism, and loss of genome integrity (Yuan et al., 2018). Furthermore, these phenotypes are reflected in RAS mutant cancer’s clinical behavior, which is associated with poorer outcomes, including decreased overall survival, bolstered by resistance to diverse chemotherapy and radiotherapy schemes (Lièvre et al., 2006; Jancík et al., 2010). Mutations in other codons of RAS are at the origin of milder conditions called RASopathies, which are characterized by distinctive craniofacial features, short stature, and learning disabilities, among other hallmarks (Mo et al., 2018).

Strategies to therapeutically target mutant RAS have met a tough road throughout the years. Approaches targeting posttranslational modifications of RAS that mediate its membrane localization or its signalling output have been overcome by the cell through multiple redundant feedback loops (Stephen et al., 2014). Moreover, in some cases, the use of more than one drug to tackle cancer cells’ feedback loops has proven prohibitively toxic (Stephen et al., 2014; Singh et al., 2015).

On the other hand, RAS’ three-dimensional conformation, which displays relatively shallow grooves, as well as its picomolar affinity for GTP/GDP, hampered the development of small molecule inhibitors (Grabocka et al., 2015; Esposito et al., 2019). However, in 2013, a new pocket was identified in KRAS that was not apparent in previous crystallographic structures (Ostrem et al., 2013). Based on this discovery, compounds were designed that covalently bind to the mutant cysteine of KRAS G12C and disrupt both switch 1 and switch 2 regions. As a consequence, KRAS G12C inhibitors thwart the GTPase’s preference to favour GDP binding over GTP, concomitantly inhibiting its signalling activity by precluding RAS interaction with RAF (Ostrem et al., 2013). Notably, since these compounds target a mutant cysteine, they spare the WT protein, underscoring their suitability as cancer therapeutic agents (McCormick, 2020). Several new covalent KRAS G12C inhibitors were rapidly developed, and this kind of molecules entered clinical trials only 6 years after the publication of the paper describing the new pocket and inhibitor (Goebel et al., 2020). One of them, AMG510, was approved in May 2021 after demonstrating an objective response rate of 36% with a median response duration of 10 months in a phase 2 trial in mutant advanced solid tumors (in combination with anti-PDL1 therapy and midazolam). AMG510 is currently commercialized by Amgen under the name Sotorasib and is evaluated in at least 13 trials. The other compound, MRTX849, is currently under scrutiny in phase 2 and phase 3 trials and was granted the breakthrough therapy designation by the FDA, which might expedite its approval.

Unfortunately, the percentage of patients that can benefit from KRAS G12C covalent inhibitors is relatively limited since this mutation represents no more than 14% of all KRAS mutations found in human tumors (Lu et al., 2016). For instance, KRAS G12C represents only 2% of all KRAS mutations in Pancreatic Ductal Adenocarcinoma (PDAC) (Kim et al., 2021). However, other efforts to target RAS GTPases are also yielding very promising results. Recent advances in screening technologies have prompted preclinical progress, resulting in the detection of RAS mutant cell vulnerabilities. One of the resulting approaches, termed synthetic lethality, consists of taking advantage of the exclusive dependence of mutant cells (RAS mutants in this case) on a second target (Singh et al., 2015). This will be discussed below for RAS, but the most commonly cited example in the literature is the therapeutic use of PARP inhibitors in BRCA1/2 mutant cancer (Marcotte et al., 2012).

RAS vulnerabilities present an extremely valuable resource for developing mutant RAS cancer therapies. In order to take advantage of this asset, it is crucial to understand the mechanisms that support mutant RAS cancer survival in the clinical setting. In the following sections, we explore the development of RAS-dependent chemotherapy and radiotherapy resistance through the DNA damage repair pathways; along the carcinogenic process, we expose mechanistic details of such resistance pathways and propose future directions of this exciting field.

## RAS in Oncogene-Induced Senescence

RAS mutation is an early event in several tissues along the multistep carcinogenic process. In fact, several mouse models have been used to demonstrate that KRAS mutation alone is sufficient to initiate tumor development (Grabocka et al., 2014). Observations in human pancreatic cancer development provide further support to mutant RAS' early contribution to carcinogenesis. RAS alterations are commonly detected in early PanIN lesions, hyperplasias that precede the development of pancreatic intraepithelial neoplasia, one of the most, if not the most, lethal solid malignancy (Luo, 2021). However, early incipient cancer cells face the struggle of surviving in extremely adverse conditions since mutant RAS constant signalling leads to replicative, metabolic, and oxidative stress (Grabocka et al., 2015). For instance, constitutively active RAS abnormally increases the formation of replication forks on replisomes and promotes the generation of asymmetric replication forks (Di Micco et al., 2006). Also, the overexpression of RAS proteins decreases cellular dNTP concentration, which forces the premature termination of replication forks. This is a consequence of the downregulation of the ribonucleotide reductase subunit M2 (RRM2), mediated by RAS proteins, leading to DNA replication stress, cell cycle stress and senescence (Di Micco et al., 2007; Rai et al., 2011). Unresolved DNA replication stress can lead to DNA damage, giving rise to several types of mutations, including chromosomal rearrangements, and DNA amplifications or deletions (Sirbu and Cortez, 2013; Zeman and Cimprich, 2014; Gaillard et al., 2015; Blackford and Jackson, 2017).

As discussed above, the replicative, oxidative, and metabolic stresses resulting from RAS mutation represent an obvious drawback for incipient cancer cell proliferation and survival. In primary cells, much of this disadvantage is mediated by Oncogene-Induced Senescence (OIS), a state of permanent cell cycle arrest in the absence of telomere erosion, that prevents the proliferation of cells in which excessive damage could lead to a full malignant phenotype (Batsi et al., 2009). Current evidence suggests that OIS is the result of constant exposure to sublethal doses of stressors (Mijit et al., 2020). Depending on the intensity of the stress, cells may exceed a threshold that promotes programmed cell death instead of senescence, although other factors, such as the cell type and the type of stimulus, may tilt the balance towards either outcome (Mijit et al., 2020).

Several different pathways activate OIS in response to RAS signalling (Mijit et al., 2020). Among them, the best understood involves the DNA Damage Response (DDR). This pathway can be activated either by exposed stretches of single-stranded DNA caused by replication fork stalling or DNA breaks resulting thereof, or by DNA damage caused by ROS. Both initiating events have been listed as natural consequences of RAS-mediated oncogenic stress. DNA damage activates ATM/ATR kinases, which stabilize p53 through phosphorylation of its serine residues 15 and 20, and by inhibitory phosphorylation of its ubiquitin ligase MDM2 (Mijit et al., 2020). In turn, p53 upregulates the cyclin-dependent kinase inhibitors p21cip1 and p16INK4A, concomitantly preventing cell cycle progression (Mijit et al., 2020).

Alternative DDR-independent mechanisms of OIS have been elucidated, including the RAS-mediated NORE1A activation. NORE1A is a recently identified downstream RAS effector which, in conjunction with the kinase HIPK2, promotes p53 pro-senescence acetylation and inhibits its pro-apoptotic phosphorylation (Donninger et al., 2015). NORE1A can also form a complex with the phosphatase PP1A and promote the activation of the cell cycle progression inhibitor Rb, by dephosphorylation (Barnoud et al., 2016).

It has also been demonstrated that RAS G12V stimulates OIS in IMR-90 non-cancerous lung fibroblasts (Batsi et al., 2009). Mechanistically, the oncogenic stress instigated by RAS G12V promotes DNA double-strand breaks and the consequential activation of the DDR. Upon DDR activation, Chk1 and Chk2 activate p53. Among its multiple effects, p53 inhibits p65, one of the two subunits that compose the transcription factor NF-kB (Mijit et al., 2020). In unstimulated cells, NF-κB is localized to the cytoplasm in a complex with its inhibitor IκBα, which prevents NF-κB translocation to the nucleus. Upon stimulation with different external signals, such as TNF-ɑ, the Iκκ complex phosphorylates IκBα, promoting its ubiquitylation and subsequent degradation. IκBα degradation allows for NF-κB translocation to the nucleus and transcriptional activity, which upregulates several genes associated with cell survival, chemotherapy and radiotherapy resistance, stromal adhesion molecules, and autocrine stimulation receptors (Xia et al., 2014). Remarkably, forced expression of the Iκκ subunit Iκκβca can relieve p53-induced inhibition of NF-κB, thereby delaying the onset of OIS (Batsi et al., 2009). Interestingly, the DDR itself promotes Iκκ activation through the action of ATM, but such endogenous activation can be overcome by wild-type p53 (Batsi et al., 2009). In fact, it has been shown in mouse embryonic fibroblasts expressing RAS G12D that p53 loss of heterozygosity (LOH) is required for sustained NF-κB nuclear localization. Furthermore, conditional p53 reactivation in human lung tumor cells has been demonstrated to restore p65 cytoplasmic localization (Meylan et al., 2009).

Once proliferating RAS mutant cells can bypass OIS, some mechanisms of genome fidelity safeguard become beneficial for cancer cell survival by mitigating the catastrophic effects of high stress levels and DNA damage (Gilad et al., 2010). This has been demonstrated for the tumor suppressor ATR, which is activated by oncogenic KRAS G12V-transformed murine embryonic fibroblasts. In this model, oncogenic transformation increases cellular reliance on the ATR-CHK2 pathway for survival. RNAi-mediated ATR targeting in p53^+/−^cells leads to p53 LOH, bolstering tumorigenesis. Interestingly, when stronger ATR silencing is achieved, cells with the same genetic background (KRAS G12V/p53^+/−^) attain intolerable levels of genomic instability, leading to decreased proliferation and cell death (Gilad et al., 2010). Similarly, a large shRNA screen performed in the colorectal cancer cell line DLD-1 identified synthetic lethality relations between RAS mutation and several components of the Base Excision Repair (BER) pathway, including NEIL2, XRCC1, Polymerase β (Pol-β), and the DNA ligase III (Luo et al., 2009). Therefore, OIS relies on the proper function of tumor suppressor genes, but tumor suppressors do not represent an eternally impervious barrier since such genes can suffer inactivating mutations and LOH. In this context, tightly regulated mechanisms of stress surveillance promote tumorigenesis.

## RAS in the Cellular Redox Balance

**FIGURE 1:**
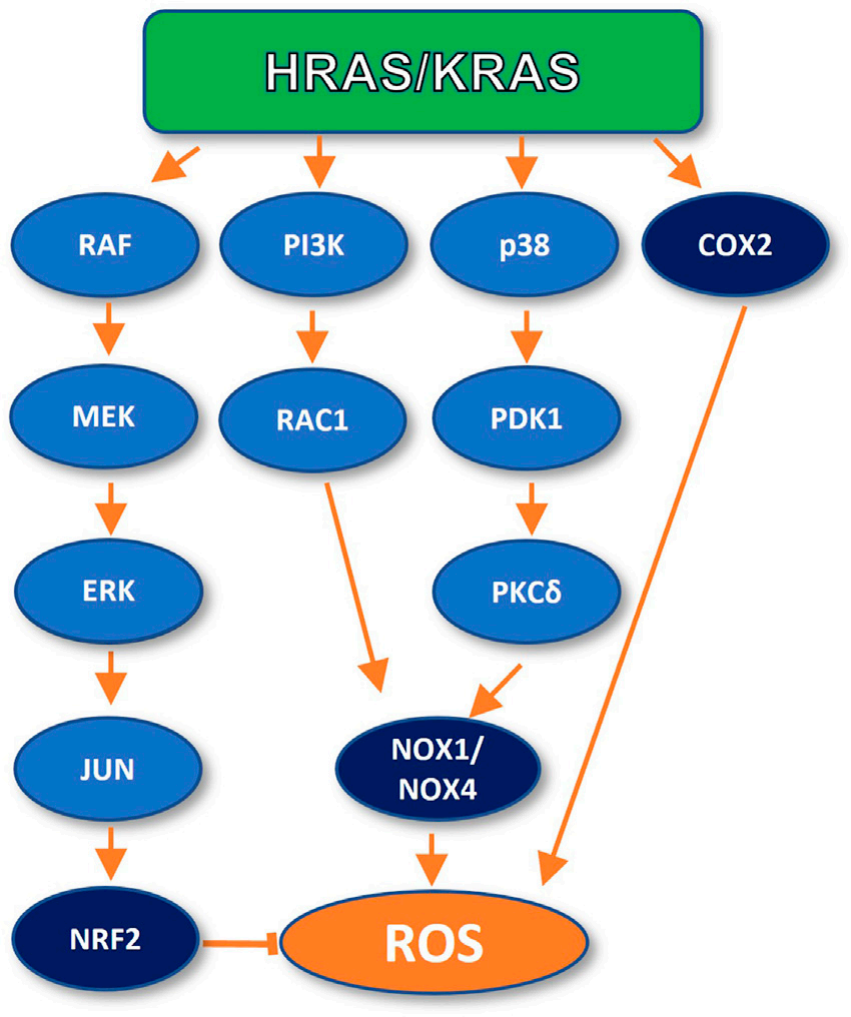
Oncogenic RAS can inhibit and promote ROS generation. In early carcinogenesis, RAS inhibits ROS production by activating the NRF2 transcription factor. However, oncogenic RAS can also promote ROS generation through upregulation of NADPH oxidases (NOX1 and NOX4) and directly by COX2 activation.

RAS has been shown to promote antioxidant as well as pro-oxidant programs in the cell (Lim and Leprivier, 2019) (Figure 1) The promotion of a RAS-dependent antioxidant response is supported by recent literature (Lim and Leprivier, 2019). It has been shown that endogenous expression of KRAS G12D in mouse embryonic fibroblasts promotes the activation of NRF2, a central player in the cellular antioxidant response, through the RAF/MEK/ERK/JUN pathway (DeNicola et al., 2011). In turn, NRF2 upregulates ROS-scavenging factors, such as Hmox1, Nqo1, Gclc, and Ggt1, to maintain the intracellular redox balance in check. Furthermore, genetic ablation of NRF2 impairs RAS-dependent tumor growth and proliferation (DeNicola et al., 2011). These findings argue for a role of RAS in limiting OIS during early tumor development.

The antioxidant response initiated by mutant RAS has also been shown to mediate chemotherapy resistance in established tumors. It has been reported that cisplatin induces mitochondrial ROS generation, increasing the stress levels present in cancer cells (Marullo et al., 2013). Platinum-based compounds like cisplatin, carboplatin, and oxaliplatin are chemotherapeutic agents widely used in cancer treatment. Such agents intercalate into DNA, interfering with RNA transcription and DNA replication by binding to N7 of guanine and adenosine residues, adduct formation, and subsequent apoptosis. However, platinum-based treatment can be overcome by cancer cells due to intrinsic resistance or acquired resistance through improved cell DNA repair and the overactivation of the anti-oxidative stress pathway (Oun et al., 2018). Tao and colleagues reported that cisplatin chemoresistance in non-small cell lung cancer cells and lung tumor tissue can be mediated by KRAS G12D-dependent activation of the transcription factor NRF2 pathway, by enhanced NRF2 mRNA expression and, therefore, increased gene expression of drug metabolizing enzymes, antioxidant enzymes, and drug transporters, thereby limiting cisplatin toxicity in cancer cells (DeNicola et al., 2011; Tao et al., 2014). Furthermore, KRAS G12C mutants were found to be less sensitive to cisplatin treatment *in vitro* and *in vivo* as a result of DNA BER stimulation, which removes cisplatin from DNA before the formation of DNA adducts (Caiola et al., 2015).

On the other hand, most of the literature concerning the impact of RAS on cellular redox balance has shown a role for RAS in the generation of ROS (Lim and Leprivier, 2019), which promote multiple phenotypes associated with cancer development, such as increased DNA oxidation (Woo and Poon, 2004; Lim and Leprivier, 2019), increased proliferation (Irani et al., 1997; Ogrunc et al., 2014), chromosome breaks with concomitant chromosomal instability (Woo and Poon, 2004), anchor-independent growth (Weinberg et al., 2010), and increased DNA-repair upon cisplatin or UV-induced insults (Cho et al., 2002). As discussed above, although some of these effects are known to trigger OIS or cell death in tumor suppressor-proficient cells, loss of tumor suppressor genes constitutes a turning point in tumor development.

RAS has also been shown to play a central role in the ROS-dependent activation of the DDR, thereby preventing extreme genomic instability levels, and promoting resistance to chemotherapy and radiotherapy-induced cell death through DNA repair.

RAS proteins can promote ROS production and consequent stimulation of DNA repair through different pathways. For instance, mutant RAS expression promotes changes in cellular metabolism, increasing the intracellular levels of hydrogen peroxide (H_2_O_2_) and Reactive Oxygen Species (ROS), promoting the oxidation of the DNA, proteins, and lipids (Lee et al., 1999). In fact, the Qo site of the mitochondrial complex III has been identified as the main site of KRAS-driven ROS generation in a mouse model of lung cancer (Weinberg et al., 2010). It has also been observed that in mouse lung cells, KRAS mutant expression promotes ROS peroxide production through cyclooxygenase 2 (COX-2) (Maciag et al., 2004). Furthermore, Park and colleagues reported that KRAS induced ROS generation through a signalling axis specifically involving the p38 MAPK in normal human fibroblasts. KRAS induced activation of p38, which led to PDPK1 activation. Once active, PDPK1 interacts with and phosphorylates PKCδ which, in turns, interacts with and phosphorylates the SH3-N domain of p47phox, a subunit of the NADPH Oxidase 1 (NOX-1). This interaction mediates p47phox membrane translocation and activation of NADPH oxidase-1 (NOX-1) upregulating cellular ROS production (Park et al., 2014). Moreover, mutant KRAS has also been shown to upregulate Nox1, a homologue of the catalytic subunit of NOX-1 at the transcriptional level, through the MAPK pathway, in normal rat kidney epithelial cells. In this study, the specific inhibitor PD98059 was used to target p38, which demonstrated the participation of such signaling cascade in ROS generation, and the enhancement of cell growth and malignant transformation (Park et al., 2014).

On the other hand, it has been observed that oncogenic HRAS expression in NIH3T3 stimulates ROS production through the HRAS/PI3K/RAC1/NADPH oxidase signaling cascade. In this study, ROS promoted DNA repair upon challenge with cisplatin and UV light-induced insults. Furthermore, pre-treatment of the cells with the antioxidant N-acetyl-cysteine partially suppressed such enhanced DNA repair (Cho et al., 2002). A similar mechanism of ROS generation was observed in normal human fibroblasts, through NOX4, in an independent analysis (Ogrunc et al., 2014).

Overall, the relation of RAS with ROS may seem confusing since some reports show that RAS signaling antagonizes ROS, while others demonstrate that it promotes ROS generation. A reconciling model proposed that RAS plays distinct, sequential roles in the cellular redox balance along carcinogenesis (Lim and Leprivier, 2019), hypothesizing that mutant RAS activates antioxidant programs upon tumorigenic initiation; then, in a more advanced carcinogenic setting, amplified RAS signaling would activate pro-oxidant programs, enhancing the cellular capacity of DNA repair and proliferation. To test this model, it will be interesting to assess the alterations associated with anti-to pro-oxidant switching in terms of genetic, epigenetic, and tumor microenvironment along carcinogenesis.

## Influence of RAS in DNA Repair Pathways

**FIGURE 2:**
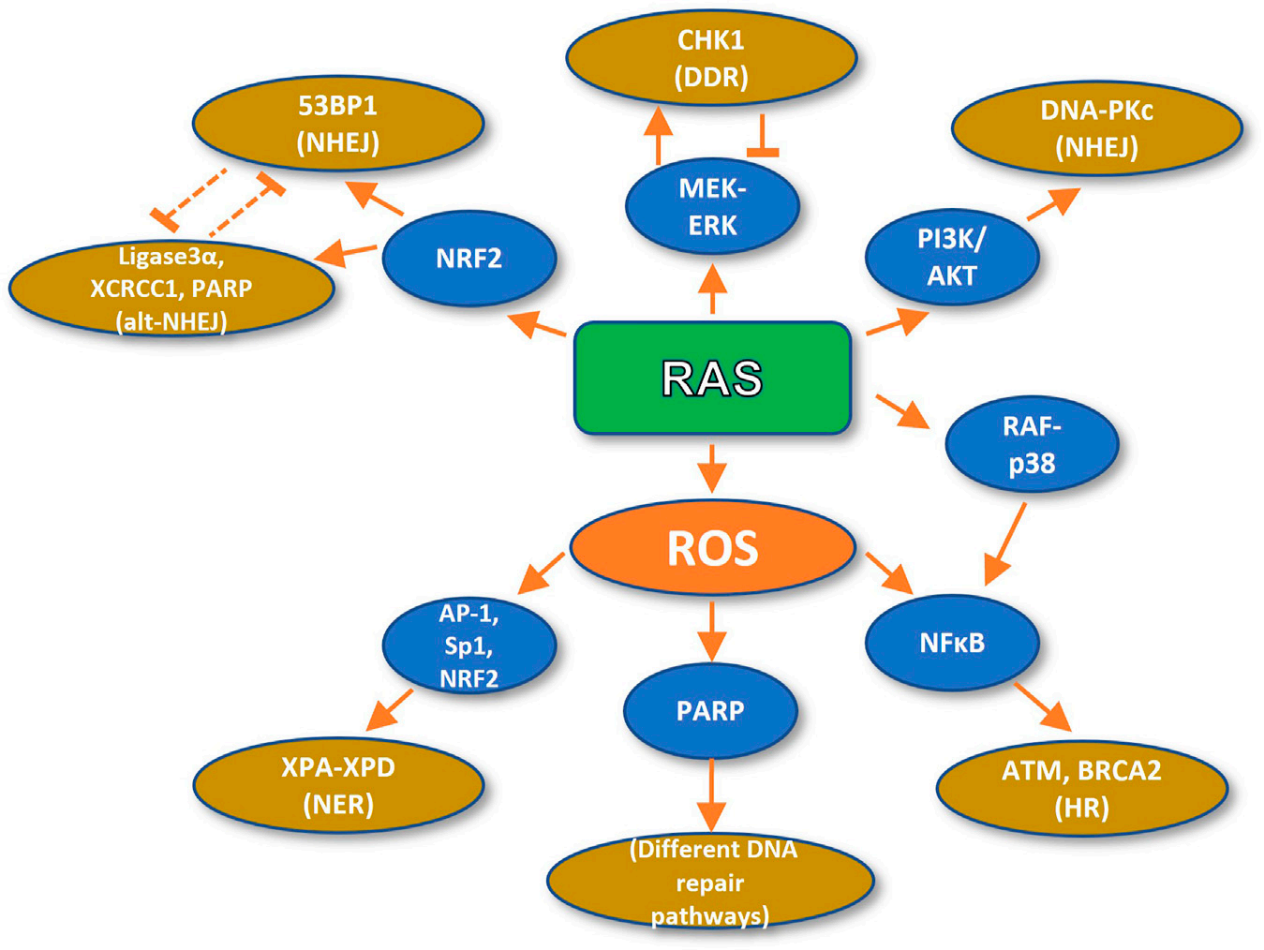
Oncogenic RAS promotes DNA repair. RAS-dependent ROS formation stimulates DNA repair (HR and NER) and the DDR by the activity of NFkB, AP-1, Sp1, and NRF2 transcription factors and PARP activation. On the other hand, RAS fosters DNA repair (NHEJ and alt-NHEJ) directly through activation of MEK, PI3K, and p38 pathways and the NRF2 transcription factor.

RAS-dependent ROS stimulate DNA repair through the activation of NF-κB, an essential mediator of chemoresistance and radioresistance which promotes DNA repair and cancer cell survival (Figure 2). It has been shown that p65 loss compromises DNA repair and genome stability. Conversely, treatment with the NF-κB activator TNF-α enhances DSB repair, but this enhancement can be inhibited by overexpression of a degradation-resistant version of the NF-κB inhibitor IκBα. Specifically, p65 stimulates the Homologous Recombination (HR) repair pathway by upregulating ATM and BRCA2 at the transcriptional level, and by inducing the formation of a BRCA1 complex with the CtIP, which is required for DSB resection, necessary for single-strand ends in the process of HR (Volcic et al., 2012).

On the other hand, high intracellular H_2_O_2_ concentrations have been shown to upregulate poly (ADP-ribose) polymerase (PARP), which is required for DNA DSB repair (Ziemann et al., 1999). Moreover, ROS activate several transcription factors, such as AP-1, Sp1, NRF2, and p53 (Cho et al., 2002). Remarkably, several genes implicated in DNA repair bear redox-sensitive transcription factor binding motifs. For instance, the promoters of the XPA, XPB, XPC, and XPD genes, implicated in nucleotide excision repair XPA-XPD contain binding sites for the aforementioned Sp1, Ets1 (member of the AP-1-like family of transcription factors), and p53 transcription factors (Cho et al., 2002).

Therefore, RAS-mediated ROS enhancement promotes the activation of DNA repair through different mechanisms. This represents a major drawback for incipient cancer cells. However, once tumor cells have overcome the proliferation-counteracting OIS induction systems, RAS-mediated ROS-dependent activation promotes cell survival by preventing intolerable genomic instability, and provide the possibility to efficiently repair radiotherapy and chemotherapy-induced DNA damage.

Besides ROS, RAS can promote DNA repair and/or chemotherapy and radiotherapy resistance through several other pathways. This was evidenced by inhibition of HRAS prenylation in rodent cells, or inhibition of HRAS farnesylation in human tumor cells, which increased their radiosensitization (Miller et al., 1993; Bernhard et al., 1996, 1998). Also, the loss of an active RAS allele leads to a significant reduction in the survival of DLD-1 and HT1080 human cell lines upon radiation (Bernhard et al., 2000). Moreover, the inhibition of the PI3K pathway leads to radiosensitization of mutant RAS expressing cells treated with the PI3K inhibitor LY294002 (Gupta et al., 2001). Furthermore, in HCT-116 human colorectal cancer cells, mutant HRAS G12V expression increases the activation of the PI3K/AKT pathway and the activity of AKT upon radiation, promoting cell survival. However, this protective effect is abolished by AKT inhibition or by dominant-negative AKT expression, leading to increased radiation cell lethality (Carón et al., 2005).

In a recent study by Tago and colleagues, NF-κB was shown to be hyperactivated upon TNF-ɑ stimulation of HRAS G12V expressing KF-8 mouse fibroblasts. NF-κB activation occurs through RAF/p38 MAPK-mediated p65 phosphorylation at serine 276 (Tago et al., 2019), which promotes NF-κB transcriptional activity. The authors of this study also reported higher levels of phosphorylated p65 in neoplastic tissue from mutant KRAS colorectal cancer samples. Furthermore, shRNA targeting of KRAS prevented the TNF-ɑ hyperstimulation of NF-κB transcriptional activity in the A549 human lung cancer cell line, as measured by the abundance of its transcriptional targets COX2, ICAM1, and A20 (Tago et al., 2019).

Moreover, mutant KRAS has also been shown to promote autocrine stimulation of the MAPK pathway through the production of EGFR ligands. In response to radiation and under such autocrine stimulation, the PI3K/AKT pathway enhances DSB repair and concomitant radioresistance through phosphorylation of serine 2056 of DNA-PKc catalytic subunit, a critical regulator of the Non-Homologous End Joining DNA repair signalling cascade (Minjgee et al., 2011).

RAS/MEK signalling is also implicated in chemotherapy and radiotherapy resistance through the activation of the DNA damage response. It has been demonstrated that RAS signalling promotes CHK1 expression in human cancer cells, and that such expression can be abolished by MEK inhibition, through treatment with the specific MEK inhibitor cobimetinib (Lee et al., 2017). Furthermore, increased RAS/MEK/ERK signalling has been associated with resistance to the CHK1 inhibitor GDC-0425 (Lee et al., 2017). Nevertheless, MEK inhibition protects cells from reduced viability upon GDC-0425 treatment. Also, CHK1 decreases ERK activation in GDC-0425-sensitive cells. As in the case of ATR mentioned before, the authors of this study interpreted the data as a feed-forward and feedback loop between RAS and CHK1 which enables neoplastic cells to maximize growth without exceeding a threshold of intolerable DNA damage (Lee et al., 2017). Similarly, another study demonstrated that the MEK inhibitor GSK1120212 radiosensitizes KRAS mutant pancreatic cancer cell lines MIAPaCa-2 and AsPC-1. Treatment with GSK1120212 delayed ɤH2AX foci disappearance and inhibited BRCA1 and RAD51 foci formation after radiation treatment. Furthermore, treatment with GSK1120212 also inhibited the disappearance of DNA-PKc and 53BP1 foci after radiation. Hence, it was concluded that MEK promotes radioresistance in pancreatic cancer cells through the activation of both the HR, and the NHEJ pathways (Poon et al., 2017). Interestingly, wild type HRAS and NRAS are also implicated in efficient Chk1 activation in mutant KRAS cells. Concordantly, the knockdown of wild type HRAS or NRAS specifically sensitizes KRAS mutant cells to DNA damaging agents (Grabocka et al., 2014).

Furthermore, it has also been demonstrated in colorectal cancer cell lines that KRAS G13D mutation can mediate radioresistance through the transcriptional upregulation of NRF2, followed by its nuclear translocation and the concomitant overexpression of 53BP1. 53BP1 translocates to the sites of DSB and promotes DNA repair through the NHEJ pathway. Interestingly, KRAS G13D was shown to accelerate DNA repair (measured by the disappearance of ɤH2AX foci) after irradiation, through the mentioned 53BP1 upregulation, while NRF2 or 53BP1 targeting radiosensitized the cells (Yang et al., 2021). Conversely, the same mutation upregulated the components of the alternative NHEJ (alt-NHEJ) pathway Ligase3α, XRCC1, and PARP1 in a different model, namely leukemic and lymphocytic cells. Interestingly, DNA repair showed delayed kinetics in response to radiation, which is a feature of alt-NHEJ (measured by the disappearance of ɤH2AX foci). Moreover, targeting alt-NHEJ components sensitized KRAS mutant cells to DNA damaging agents (Hähnel et al., 2014).

Furthermore, thymocytes derived from KRAS G12D knock in mice were shown to display increased repair through the alt-NHEJ pathway upon DNA damage with chemical agents or radiation, which was associated with an increased expression of Ligase3α, XRCC1, and PARP1 (Hähnel et al., 2014). The authors of this report proposed that the overexpression of alt-NHEJ components outcompeted classical NHEJ factors for DNA binding. Again, in a different tissue of the same animal (mouse embryonic fibroblasts) this mutation has been shown to upregulate NRF2, which, as mentioned above, promotes the NHEJ pathway through 53BP1 (DeNicola et al., 2011).

These results suggest that oncogenic RAS may have a distinct influence on the DSB repair pathway preference in tissues of different origins, underlying differences in clinical history and treatment response observed in hematological and solid neoplasms. Such differences could help guide the search for synthetic lethal interactions in cancers of different origins.

## Future Perspectives and Concluding Remarks

Despite recent advances in targeting mutant RAS tumors, this field still faces important challenges. For example, G12C targeting with the recently approved covalent inhibitor Sotorasib is very specific for the mutant protein, but this brilliant approach’s high selectivity comes at the price of benefiting a relatively small percentage of patients, as previously mentioned (Hansen et al., 2018; Kim et al., 2021). Therefore, new strategies are required to either attack RAS mutant cancer vulnerabilities or to develop new ways to directly target RAS itself.

Advances have been achieved in tackling RAS vulnerabilities by exploiting a recently discovered co-dependence between mutant KRAS and the component of the alternative NHEJ pathway PARP1. Interestingly, PARP1 is upregulated upon KRAS mutation (Hähnel et al., 2014) and, on the other hand, PARP1 resistance arises through the overactivation of RAS-MEK-ERK signaling (Sun et al., 2017). Thus, Sun and colleagues treated different types of tumor cells with combinations of MEK1/2 and PARP inhibitors both *in vitro* and *in vivo* and revealed a synergistic effect of these two kinds of drugs, specifically in KRAS mutants (Sun et al., 2017). Furthermore, their results prove that this synergy is associated with the overexpression of the transcription factor FOXO3a, which concomitantly promotes downregulation of the DDR components RAD51, BRCA1, and MRE11, while it promotes the upregulation of the proapoptotic factor BIM (Sun et al., 2017). As a result of the success obtained in the preclinical setting, a phase 1/2 clinical trial is now being conducted to test the efficacy of the combination of the two previously approved drugs Selumetinib (MEK1/2 inhibitor) and Olaparib (PARP1 inhibitor) in the treatment of ovarian and other solid malignancies with RAS pathway alterations (NCT03162627) (Sun et al., 2020).

Other notable efforts are aiming to inhibit components of the DDR chemically. Such is the case of a newly developed molecule (referred to as compound 14), that inhibits Pol-β (Yuhas et al., 2021). Pol-β is an essential component of the BER pathway which was previously shown to maintain a synthetic lethal relation with KRAS G13D in an RNAi screen. Compound 14 irreversibly inhibits the ability of Pol-β to bind to the DNA by covalently targeting two lysine residues while sparing other DNA polymerases. Remarkably, treatment with pro-14 (a prodrug derived from compound 14) promoted very low toxicity but could potentiate the cytotoxic effects of DNA damaging agents in mouse embryonic fibroblasts and HeLa cells (Yuhas et al., 2021). It will be interesting to test the ability of this new inhibitor to kill KRAS mutant cells as a mono-therapy (since Pol-β has been shown to be synthetic lethal with mutant KRAS), and to determine if this is a viable therapeutic strategy.

A different flourishing area in the RAS targeting endeavor involves RNA technology. RNAi against KRAS G12D, the most common RAS mutation in human cancer, holds the promise of very high specificity and efficient tumor killing. Recent advances in RNA delivery *in vivo* have prompted this approach to clinical trials. One of the studies is a phase 1/2a clinical trial in which a small biodegradable polymeric device directly implanted in locally advanced pancreatic tumors was used to slowly administer siRNAs against KRAS G12D over 4 months, with concomitant chemotherapy with DNA damaging agents. The treatment was shown to be safe and well tolerated, and 10/12 patients showed stable disease, and 2 showed partial response (Golan et al., 2015). These results fostered a still ongoing multinational phase 2 trial (NCT01676259) to determine the progression-free survival in patients with locally advanced pancreatic tumors receiving the treatment described above.

In another phase 1 clinical trial currently in progress (NCT03608631), exosomes containing siRNAs against KRAS G12D are being administered intraperitoneally to patients with metastatic pancreatic cancer. These exosomes are engineered to bear the CD47 surface protein, which helps to avoid clearance by monocytes, therefore increasing the stability of exosomes. This study relies on encouraging preclinical data in which these engineered exosomes showed a remarkable ability to suppress pancreatic cancer and significantly increase survival in mice when administered intraperitoneally (Kamerkar et al., 2017).

However, RNAi is not the only RNA system with potential clinical applications. Circular RNAs (circRNAs) are also capable of controlling the fate of mutant RAS cancer cells. This type of RNA was discovered several decades ago, but only recently started drawing researchers’ attention (Kristensen et al., 2021). These transcripts consist of one or multiple exons of a coding gene covalently circularized in a process known as back-splicing (Kristensen et al., 2021). Interestingly, circRNAs can control several cellular events through their interaction with RNA-binding proteins, microRNAs, or with their genomic parent locus. It has been hypothesized that circRNAs are part of an RNA interaction network and compete with mRNAs for microRNA binding (Salmena et al., 2011). Therefore, circRNAs can, for instance, increase mRNA abundance by outcompeting mRNAs for microRNA binding. The regulation exerted by circRNAs can occur both in cis and in trans, but regulation in cis is expected to be quite common because both mRNAs and circRNAs can share microRNA Response Elements (MREs), since they are transcribed from the same gene.

Recent work has demonstrated interesting links between circRNA, the DDR, and oncogenic RAS. Experimental and bioinformatic evidence support the transcription of circRNAs from several DDR genes, including ATM, ATR, CHK1, CHK2, TP53BPP1, NBS1, MRE11, RAD50, and SMARCA5 (Papaspyropoulos et al., 2021). For most of these circRNAs, the microRNA targets remain to be validated, but the role of circSMARCA5 was recently elucidated. The SMARCA5 protein is a member of the SWI/SNF complex, a chromatin remodeler necessary for the recruitment of DDR components. Specifically, SMARCA5 promotes H2AX phosphorylation and ubiquitylation in response to DNA damage, and it is overexpressed in prostate and hepatic cancer (Xu et al., 2020). Conversely, circSMARCA5, the circular RNA produced from the SMARCA5 gene, is downregulated in the prostate, hepatic, and breast cancer. Mechanistically, circSMARCA5 interacts with its parent locus on the genomic DNA and promotes premature termination of the circSMARCA mRNA, ultimately leading to a truncated nonfunctional protein. Therefore, circSMARA5 expression indirectly decreases the DNA repair capacity, consequently increasing sensitivity to the DNA damaging agents cisplatin and bleomycin (Xu et al., 2020).

Interestingly, mutant KRAS decreases the expression of a large number of circRNAs, including circSMARCA5 (Dou et al., 2016). This kind of interactions could be exploited to target the DDR in cancer cells using novel RNA *in vivo* delivery methods to administer DDR-hindering RNAs such as circSMARCA5 in combination with DNA damaging agents. An advantage of RNA-based treatments is that different transcripts could be delivered at once, and tumor RNA profiles could be used to personalize RNA cocktails.

It is worth mentioning that therapies that target the DDR take advantage of the exacerbated genomic stress of RAS mutant tumors, leading to intolerable levels of genomic instability and subsequent cell death. Therefore, a possible strategy could consist of first specifically targeting the DDR in combination with genotoxic agents and then using RAS-inhibiting molecules to overcome resistance to DDR inhibitors and genotoxic agents, since resistance to treatment arises very fast in many RAS mutant cancers, including PDAC (Amrutkar and Gladhaug, 2017). Experimental testing should challenge this speculative rationale.

Mutations of the RAS oncogenes have a profound impact on multiple aspects of the cell. Their effects are so diverse that the literature has met controversies around the participation of RAS in cell biology. Such is the case of its impact on the cellular redox balance and association with stress and DNA damage surveillance mechanisms. However, a comprehensive understanding of the diverse mutant RAS effects in the context of the carcinogenic process will help solve such controversies, ultimately leading to solid foundations upon which new treatments could arise.
